# Study on Tissue Homogenization Buffer Composition for Brain Mass Spectrometry-Based Proteomics

**DOI:** 10.3390/biomedicines10102466

**Published:** 2022-10-02

**Authors:** Adam Aleksander Karpiński, Julio Cesar Torres Elguera, Anne Sanner, Witold Konopka, Leszek Kaczmarek, Dominic Winter, Anna Konopka, Ewa Bulska

**Affiliations:** 1 Faculty of Chemistry, University of Warsaw, Pasteura 1, 02-093 Warsaw, Poland; 2 Nencki Institute of Experimental Biology of the Polish Academy of Sciences, Pasteura 3, 02-093 Warsaw, Poland; 3 Biological and Chemical Research Centre, Faculty of Chemistry, University of Warsaw, Żwirki i Wigury 101, 02-089 Warsaw, Poland; 4 Institute for Biochemistry and Molecular Biology (IBMB), Medical Faculty, University of Bonn, Nußallee 11, 53115 Bonn, Germany; 5 Lukasiewicz Research Network—PORT Polish Center for Technology Development, Stablowicka 147, 54-066 Wroclaw, Poland; 6 Nencki-EMBL Center of Excellence for Neural Plasticity and Brain Disorders: BRAINCITY, Nencki Institute of Experimental Biology of the Polish Academy of Sciences, Pasteura 3, 02-093 Warsaw, Poland

**Keywords:** proteomic analysis, brain proteomics, sample preparation

## Abstract

Mass spectrometry-based proteomics aims to study the proteome both qualitatively and quantitatively. A key step in proteomic analysis is sample preparation, which is crucial for reliable results. We investigated the effect of the composition of the homogenization buffer used to extract proteins from brain tissue on the yield of protein extraction and the number and type of extracted proteins. Three different types of buffers were compared—detergent-based buffer (DB), chaotropic agent-based buffer (CAB) and buffer without detergent and chaotropic agent (DFB). Based on label-free quantitative protein analysis, detergent buffer was identified as the most suitable for global proteomic profiling of brain tissue. It allows the most efficient extraction of membrane proteins, synaptic and synaptic membrane proteins along with ribosomal, mitochondrial and myelin sheath proteins, which are of particular interest in the field of neurodegenerative disorders research.

## 1. Introduction

Global proteomics aims to examine all proteins in a given tissue using various analytical methods. Currently, mass spectrometry (MS) is a gold standard for studying the proteome both qualitatively and quantitatively. A commonly used strategy in MS-based proteomics, the so-called bottom-up strategy, involves proteolytic cleavage of proteins to generate peptides, which are further analysed by mass spectrometry. Advances in chromatographic separation of enzymatically cleaved peptides and their subsequent detection by tandem mass spectrometry have made it possible to identify thousands of proteins within a single-shot LC-MS/MS experiment [1,2]. Global comparative proteome analyses of healthy and diseased tissues can provide valuable information on the specific pathological mechanism of a given disease [3,4,5,6,7]. This knowledge can support the development of targeted therapies [8] or diagnostic tests based on identified biomarkers [3,9].

Sample preparation is the first step of any MS-based tissue proteomic workflow, and therefore crucial to produce reliable results. Generally, it involves tissue homogenisation, subsequent protein extraction, disulphide bond reduction, sulfhydryl group alkylation, enzymatic protein hydrolysis and solid-phase extraction-based desalting of obtained peptides. Sample preparation can be performed according to various procedures, which include: in-gel- and in-solution-based protein digestion, filter-aided sample preparation (FASP) [10], suspension trapping filter-based sample preparation (S-trap) [11] and single-pot, solid-phase-enhanced sample preparation (SP3) [12].

Sample preparation is particularly important when processing brain tissue samples, which have a high lipid content that can severely interfere with chromatographic separation of peptides derived from enzymatically cleaved proteins. In this case, using the in-solution digestion procedure, additional protein-precipitation steps to concentrate and separate proteins from other cellular constituents i.e., lipids, nucleic acids, fatty acids, etc., are necessary and beneficial to obtain good quality results. Chloroform/methanol [13], acetone [14] and trichloroacetic acid [15] are frequently used as protein-precipitating reagents. Subsequent solubilisation of precipitated proteins can be achieved by using chaotropic agents such as urea [16], thiourea and acid-labile detergents such as sodium deoxycholate [17] or RapiGest^TM^ [16,18]. Once the protein precipitate is dissolved, further steps of disulphide bridges’ reduction and alkylation of free SH groups are carried out, followed by enzymatic digestion. Trypsin is the most widely used proteolytic enzyme.

In global differential proteomic analyses, there is a need to maximize the number of identified proteins to cover the studied proteome as broadly as possible. Since the composition of the homogenization buffer strongly affects the number of protein identifications by mass spectrometry, the selection of optimal tissue homogenization buffer is crucial, and its composition should be optimized.

The aim of this study was to optimize sample preparation, in particular the composition of the tissue homogenization buffer applied to a proteomic approach with in-solution digestion for use in brain tissue proteome analyses regarding protein extraction yield and number of identified proteins. Already, information obtained from mass spectrometry-based proteomic measurements of brain tissue is helping to understand the pathomechanism of neurodegenerative diseases such as Alzheimer’s disease [19,20] and TDP-43 proteinopathies i.e., amyotrophic lateral sclerosis [21] and fronto-temporal lobar degeneration [22]. The resulting optimized protocol may be applied for global comparative proteomic profiling of human brain tissue collected post-mortem from patients affected by TDP-43 proteinopathies i.e., amyotrophic lateral sclerosis (ALS) and fronto-temporal lobar degeneration (FTLD).

## 2. Materials and Methods

### 2.1. Reagents

LC-MS-grade water, acetonitrile, methanol, dimethyl sulfoxide (DMSO) and acetone were purchased from EMD Millipore (Burlington, MA, USA). Formic acid, trifluoroacetic acid, triethylammonium bicarbonate (TEAB), Tris-(hydroxymethyl)-aminomethane hydrochloride (Tris-HCl), dimethyl sulfoxide (DMSO), 2-[4-(2-hydroxyethyl)piperazin-1-yl]ethanesulfonic acid (HEPES), urea, thiourea, sucrose, NaCl, 2,2′,2″,2‴-(Ethane-1,2-diyldinitrilo)tetraacetic acid (EDTA), sodium dodecyl sulfate (SDS), dithiothreitol (DTT) and acrylamide were purchased from Sigma-Aldrich (St. Louis, MO, USA). RapiGest was obtained from Waters (Milford, MA, USA). Mass spectrometry grade trypsin was purchased from Promega (Madison, WI, USA). Protease inhibitor (complete mini EDTA-free protease inhibitor cocktail containing 4-(2-aminoethyl) benzenesulfonyl fluoride hydrochloride (AEBSF)) and phosphatase inhibitors (containing sodium orthovanadate and cantharidin) were obtained from Roche (Basel, Switzerland).

### 2.2. Tissue Collection

All MS analyses were conducted on tissue derived from whole rat brain in accordance with the Polish Act on Animal Welfare. Animals (female Wistar rats) were sacrificed, their brains were rapidly isolated, snap-frozen and stored at −80 °C until further preparation.

### 2.3. Sample Preparation

Homogenates were prepared in three biological replicates per each studied homogenization buffer. Each brain was homogenised in an ice-cold homogenization buffer for 60 s using an IKA T10 basic Ultra-Turrax^®^ homogenizer (IKA, Königswinter, Germany). The ratio of buffer to the tissue was 10:1 (mL/g) (v/m). The tip of the homogeniser was rinsed with methanol, water, and a given homogenization buffer before each homogenisation. The homogenate was then centrifuged at 20,000× *g* for 30 min at 4 °C (5804/5804 R Centrifuge, Eppendorf, Enfield, CT, USA). Next, the supernatant was transferred into a new Eppendorf tube. Protein concentrations were determined by the fluorescent-based protein quantitation assay (Qubit^TM^ Protein Assay Kit, Thermo Fisher Scientific, Waltham, MA, USA). Prepared homogenates were immediately frozen and stored at −80 °C. In this study the sample was thawed once to precipitate the proteins.

Samples were prepared in two technical replicates per each biological replicate (six samples per buffer). A volume of each homogenate, equal to 100 μg, was diluted with LC-MS grade water to a final volume of 100 μL. Next, 100 μL of 1 M NaCl and four volumes (800 μL) of ice-cold acetone were added. The obtained mixture was vortexed for 15 s and incubated overnight at −20 °C, then centrifuged at 20,000× *g* for 30 min at 4 °C. The supernatant was discarded; the pellet was washed twice with ice-cold methanol and air-dried. Next, the pellet was subjected to solubilisation in RapiGest (0.1%) and TEAB (100 mM, pH 8.5). The protein pellet was incubated at 37 °C for 45 min at 850 rpm (Eppendorf Comfort Thermomixer, Eppendorf, Enfield, CT, USA). To reduce disulphide bonds, DTT (10 mM) was added to the protein solution and samples were incubated at 56 °C for 60 min at 850 rpm. To alkylate cysteine residues, acrylamide (30 mM) was added to the protein solution; samples were incubated at room temperature for 25 min [23]. Alkylation was quenched by addition of an equal-molar amount of DTT and incubation at RT for 15 min. Proteins were digested overnight at 37 °C by trypsin (protein:enzyme (*w*/*w*) ratio—100:1). Digested peptides were desalted using Pierce^TM^ Peptide Desalting Spin Columns (Thermo Scientific, Waltham, MA, USA), according to manufacturer’s protocol. Desalted peptides were vacuum dried at room temperature (SpeedVac Concentrator Plus, Eppendorf, Enfield, CT, USA) and re-suspended in 100 μL of 5% acetonitrile, 0.1% formic acid and analysed by LC-MS/MS.

### 2.4. LC-MS/MS Analysis

Liquid chromatography-tandem mass spectrometry analysis was performed using a nanoflow UHPLC instrument (EASY nLC 1000, Thermo Fisher Scientific, Waltham, MA, USA) coupled on-line to an Orbitrap Velos^TM^ mass spectrometer (Thermo Fisher Scientific, Waltham, MA, USA). One microgram of peptides was separated on a reverse-phase 30-cm long C_18_ in-house packed column (100 μm inner diameter packed with 5 μm ReproSil-Pur 120 C18-AQ (Dr Maisch, Ammerbuch, Germany)). Solvent A consisted of 0.1% formic acid, 5% DMSO in water; solvent B consisted of 0.1% formic acid, 5% DMSO in acetonitrile. The following gradient elution was used: 0 min—1% B, 120 min—35% B, 121 min—95% B, 124 min—1% B, 127 min—95% B, 130 min—1% B. The total time of analysis was 140 min. The flow rate of mobile phase was set to 400 nL/min. The eluted peptides were ionized in the positive ion mode in the nano-ESI source with a capillary voltage of 1.9 kV. Mass spectrometric analysis was performed in the data-dependent acquisition mode, with dynamic exclusion for 60 **s**. Survey scans from 350 *m/z* to 1600 *m/z* were acquired by Orbitrap mass analyser at a resolving power of 60 000. CID-MS/MS spectra of the top 10 most abundant, multiply charged ions were performed in the ion trap.

### 2.5. Data Analysis

Statistical analysis for both extraction efficiency differences and differences in number of identified proteins was conducted in Excel (Microsoft, Redmond, WA, USA) with XLSTAT add-on (one-way ANOVA with Tukey post-hoc test).

MS data was analysed by FragPipe (v. 17.1) (Nesvilab, University of Michigan, Ann Arbor, MI, USA) with MSFragger (v. 3.4) [24] and Philosopher (v. 4.2.1) [25]. Raw spectra files were converted to mzML using ProteoWizard’s MSConvert (v. 3.0.1908) (Palo Alto, CA, USA) with vendor’s peak picking [26]. Spectra were searched against SwissProt *Rattus Norvegicus* (UP000002494) database (canonical and isoform sequences; 9778 entries; downloaded on 29 March 2022). The following parameters were set: trypsin as digestion enzyme in semi-specific mode; precursor and fragment ion mass tolerance—10 ppm and 0.6 Da, respectively. Mass calibration and parameter optimization were disabled. The isotope error was set to 0/1/2. Propionamidation of cysteine was set as fixed modification; oxidation of methionine and deamidation of asparagine and glutamine were variable modifications; up to two missed cleavage sites were allowed. The peptide length was set from 6 to 35, the peptide mass was set in the range from 500 Da to 5 000 Da. Proteins and peptides were identified using the target-decoy approach with reversed database. Philosopher [25] with PeptideProphet [27] and ProteinProphet [28] was used to estimate the identification false discovery rate (FDR). Results were processed with FDR set to 1% at the PSM, peptide and protein levels. PeptideProphet minimal probability was set to 0.90. Quantification of proteins was performed using IonQuant [29]. The minimal ratio count was 2, the MaxLFQ [30] algorithm and match between runs (MBR) with 3 donor runs and 0.5 minimal correlation ratio were used for quantification. Ion-, peptide-, and protein-level MBR FDR thresholds were all set to 1%.

Statistical analysis was performed with Perseus (v. 2.0.3) (Max Planck Institute of Biochemistry, Martinsried, Germany) [31]. Only proteins with valid quantitative information present in 4 of 6 samples in at least one group were used for statistical testing in label-free relative quantitative analysis. Missing values were replaced based on quantile regression imputation of left-censored data (QRILC) available in Perseus through the PerseusR plugin [32]. The permutation-based FDR-controlled student T-test was used for statistical analysis of differently expressed proteins between each pair of homogenates. Proteins were considered to be differentially represented if the difference in abundance was statistically significant (FDR adjusted *p*-value < 0.05) and fold change was equal to or higher than 1.

Gene ontology analysis was performed using STRING (v. 11.5) [33] (string-db.org) and the Gene Ontology Resource tool available through Panther DB [34] (geneontology.org). The minimum required interaction score in STRING was set to 0.7 (high confidence), all interaction sources were used i.e., text mining, experiments, databases, co-expression, neighborhood, gene fusion and co-occurrence. Only protein networks with protein-protein interaction enrichment *p*-value of <0.01 were considered for further analysis. Clustering was performed in accordance with the Markov clustering (MCL) algorithm [35], the MCL inflation parameter was set to 3. For each cluster only functional enrichments with an FDR of <0.01 were considered.

## 3. Results

To study the effect of tissue homogenization buffer composition on the yield of protein extraction and the number of protein identifications, three different buffers, listed in Table 1: detergent buffer (DB), chaotropic agent buffer (CAB) and detergent-free buffer (DFB), were used. The SDS used in the DB buffer is a water-soluble anionic detergent that enables dispersion of hydrophobic proteins (including membrane proteins) into aqueous solution. Moreover, SDS provides protein denaturation. Urea and thiourea, present in the CAB buffer, are small-molecule chaotropic agents with the ability to interact with both polar and non-polar parts of proteins, causing their efficient denaturation [36]. On the contrary, DFB buffer does not contain detergents or chaotropic agents, so it can be expected that mainly water-soluble cytosolic proteins will be extracted efficiently.

Rat (*Rattus norvegicus*) brain tissue was used to carry out optimisation experiments. This species is often used to model certain features of human neurodegenerative diseases [37] due to genome/proteome similarity and more advanced neurophysiology compared to mice [38].

**Figure 1:**
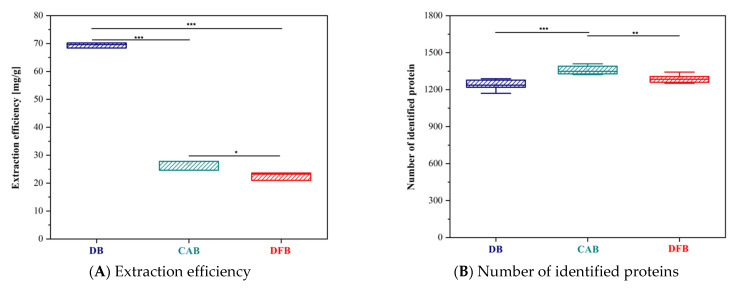

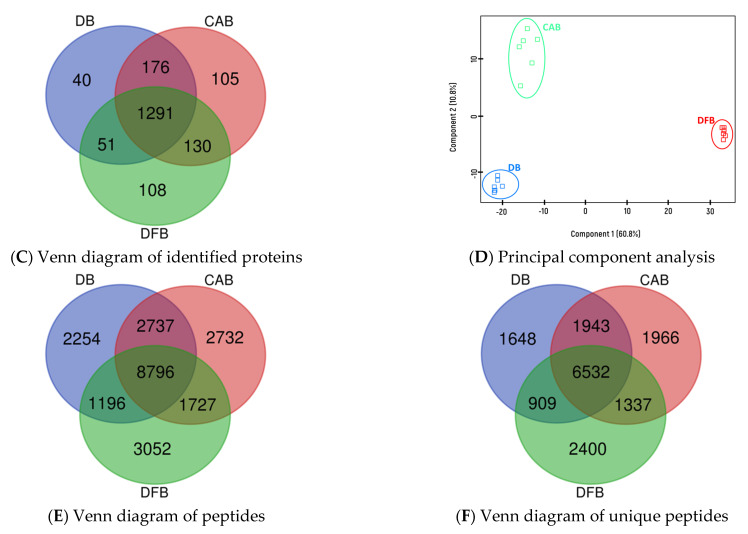
(**A**) Extraction efficiency. Box plot presenting results of protein extraction efficiency (*** *p* < 0.001; * *p* < 0.05); (**B**) Number of identified peptides. Box plot presenting number of proteins identified (*** *p* < 0.001); ** *p* < 0.01); (**C**) Venn diagram of identified peptides. Venn diagram showing the number of proteins identified, and the number of proteins characteristic for each type of homogenate; (**D**) Principal component analysis; (**E**) Venn diagram of peptides. Venn diagram presenting number of peptides identified, and the number of peptides characteristic for each type of homogenate; (**F**) Venn diagram of unique peptides. Venn diagram presenting number of unique peptides identified, and the number of unique peptides characteristic for each type of homogenate.

The highest protein extraction efficiency (Figure 1A) (approx. 3× higher compared to other buffers), calculated as the ratio of mass of proteins that were extracted [mg] to the mass of brain tissue [g], was achieved when using detergent buffer (DB), the differences were statistically significant. MS analysis allowed for the identification of 1901 proteins in total across the three different types of homogenates. The highest number of protein identifications was obtained for the CAB homogenate (Figure 1B). There were 1702 proteins identified in the CAB homogenate (1358 in each replicate on average, RSD = 2.6%); 1580 proteins were identified in the DFB homogenate (1288 in each replicate on average, RSD = 2.6%) and 1558 proteins were identified in the DB homogenate (1238 in each replicate on average, RSD = 3.4%). Statistical analysis revealed that the differences in the number of identified proteins were statistically significant between pairs: CAB vs. DB and CAB vs. DFB. There was, however, no statistically significant difference for DB vs. DFB. A total number of 1291 identified proteins were common for all three homogenization buffers, while the following numbers of unique proteins were identified for individual homogenization buffers: 105 for CAB, 108 for DFB and 40 for DB (Figure 1C). The proteomic profile of each homogenate was distinct and allowed for the differentiation and grouping of samples based on the type of homogenization buffer (Figure 1D), which was shown by PCA analysis. The highest number of peptides (Figure 1E) and unique peptides (Figure 1F) were obtained in the CAB homogenate; however, the values were not significantly different. There were 15,992 peptides and 11,778 unique peptides identified in the CAB homogenate; 14,771 peptides and 11,178 unique peptides identified in the DFB homogenate and 14,983 peptides and 11,032 unique peptides identified in the DB homogenate.

In order to categorize identified proteins, gene ontology (GO) analysis was performed. Proper brain functioning is based on the connectivity between neurons within the network. Therefore, it was reasonable to select one of the categories that covered synaptic proteins. For synaptic transmission, membrane-associated proteins which include neurotransmitter receptors, ion channels and transporters, are of particular importance. For this reason, the category of membrane proteins and membrane synaptic proteins was additionally selected. Cytosolic proteins were added as a general contrast category. The highest number of membrane proteins, synaptic proteins and synaptic membrane proteins were identified in CAB (843, 454 and 100, respectively) compared to DB (773, 434 and 90, respectively) or DFB (729, 416 and 77, respectively) homogenates. Conversely, the highest number of cytosolic proteins was identified in DFB (662) in comparison to CAB (641) and DB (600) homogenates. Data is presented in Table 2.

**Figure 2:**
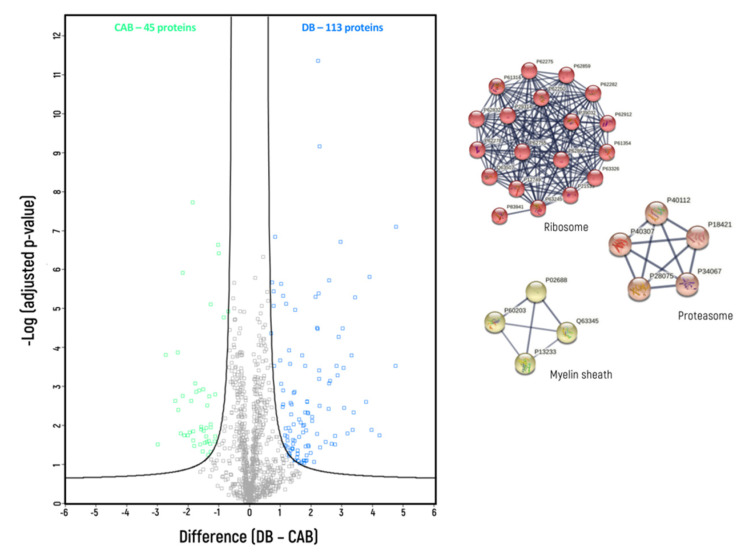
Volcano plot of proteins for which different levels were found between DB and CAB homogenization buffers (blue—overrepresented in DB buffer; green—overrepresented in CAB buffer); indicated are protein clusters which were identified by GO analysis with application of Markov clustering method (FDR < 0.01).

**Figure 3:**
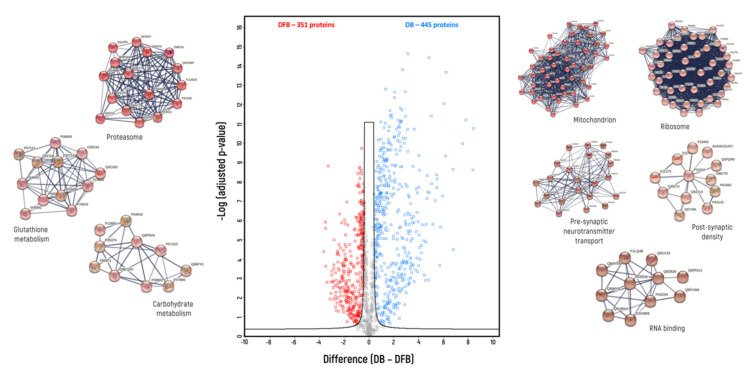
Volcano plot of proteins for which different levels were found between DB and DFB homogenization buffers (blue—overrepresented in DB buffer; red—overrepresented in DFB buffer); indicated are protein clusters which were identified by GO analysis with application of Markov clustering method (FDR < 0.01).

**Figure 4:**
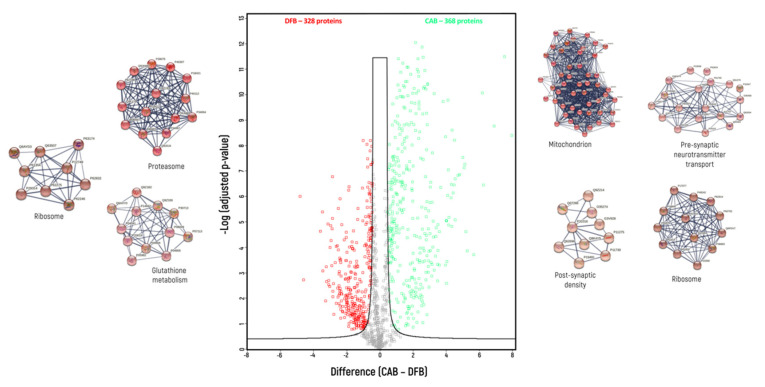
Volcano plot of proteins for which different levels were found between CAB and DFB homogenization buffers (green—overrepresented in CAB buffer; red—overrepresented in DFB buffer); indicated are protein clusters which were identified by GO analysis with application of Markov clustering method (FDR < 0.01).

Label-free protein quantification allows for the identification of proteins that had a higher abundance in each homogenate compared to the others. The comparisons were conducted pairwise—DB vs. CAB (Figure 2), DB vs. DFB (Figure 3) and CAB vs. DFB (Figure 4).

When DB and CAB were compared (Figure 2), 113 proteins were overrepresented in DB and 45 proteins were found to be overrepresented in CAB. Based on GO categories, DB vs. CAB buffer-enriched proteins comprised of 64 vs. 16 membrane proteins, 29 vs. 4 synaptic proteins, 4 vs. 0 membrane synaptic proteins and 43 vs. 16 cytosolic proteins (Table 3). Additionally, based on MCL clustering, three distinct clusters were identified among the 113 proteins overrepresented in the DB homogenate, and no distinctive clusters among the 45 proteins overrepresented in the CAB homogenate. These three DB clusters were annotated as ribosomal proteins (1st cluster—19 proteins), proteasomal proteins (2nd cluster—5 proteins), and myelin sheath proteins (3rd cluster—4 proteins).

When DB and DFB were compared (Figure 3), 455 proteins were found to be overrepresented in the DB homogenate, while 351 proteins were more abundant in the DFB homogenate. Based on GO categories, DB vs. DFB buffer-enriched proteins comprised of 301 vs. 112 membrane proteins, 170 vs. 50 synaptic proteins, 45 vs. 5 membrane synaptic proteins and 137 vs. 198 cytosolic proteins (Table 3). Based on MCL clustering, five distinct clusters were identified among the 455 proteins overrepresented in the DB homogenate, and three distinct clusters among the 368 proteins overrepresented in the DFB homogenate. These five clusters found for the DB homogenate were annotated as mitochondrial proteins (1st cluster—50 proteins), ribosomal proteins (2nd cluster—44 proteins), pre-synaptic proteins involved in neurotransmitter transport (3rd cluster—22 proteins), post-synaptic density proteins (4th cluster—12 proteins) and nuclear RNA-binding proteins (5th cluster—11 proteins). Three clusters found for the DFB homogenate were annotated as proteasomal proteins (1st cluster—16 proteins), cytosolic proteins involved in glutathione metabolism (2nd cluster—12 proteins) and cytosolic proteins involved in carbohydrate metabolism (3rd cluster—10 proteins).

When CAB and DFB were compared (Figure 4), 368 proteins overrepresented in the CAB homogenate and 328 proteins overrepresented in the DFB were determined. Based on GO categories, CAB vs. DFB buffer enriched proteins comprised of 238 vs. 103 membrane proteins, 129 vs. 49 synaptic proteins, 41 vs. 3 membrane synaptic proteins and 95 vs. 171 cytosolic proteins (Table 3). Based on MCL clustering, four distinct clusters were identified among the 368 proteins overrepresented in the CAB homogenate, and three distinct clusters among the 328 proteins overrepresented in the DFB homogenate. These four clusters found for CAB homogenate were annotated as mitochondrial proteins (1st cluster—50 proteins), pre-synaptic proteins involved in neurotransmitter transport (2nd cluster—19 proteins), ribosomal proteins (3rd cluster—13 proteins) and post-synaptic density proteins (4th cluster—10 proteins). Three clusters found for DFB homogenate were annotated as proteasomal proteins (1st cluster—15 proteins), cytosolic proteins involved in glutathione metabolism (2nd cluster—12 proteins) and ribosomal proteins (3rd cluster—9 proteins).

## 4. Discussion

Proteomic laboratories around the world use their own analytical protocols for protein extraction, which vary in detail, depending on the target tissue, cell type, cell compartment, or set of proteins of interest. Currently, there is no universal laboratory procedure which can be applied to study entire proteomes for every possible biological target.

In this work, the effect of brain tissue homogenization-buffer composition on the protein-extraction yield and numbers and types of identified proteins was evaluated. Comparison of the obtained results and their discussion in relation to literature data is problematic, because the measurements were performed on different mass spectrometers with different performance, which significantly affects the number of identifications. Additional sample preparation steps, such as protein prefractionation and peptide fractionation also increase the number of protein identifications [39]. Many search engine parameters such as peptide mass tolerance can be set in a wider or narrower range affecting resulting protein identifications [40]. It should also be noted that different structures of the same tissue such as cerebral cortex, amygdala, striatum or hippocampus within the brain, can provide different numbers of identified proteins due to different gene expression and complexity of tissue matrix [41,42]. Considering all of the above, comparing the effect of altered conditions of any stage of proteomic workflow on the absolute protein identification numbers should be performed for results obtained within the same laboratory, measurements, and identification settings, etc. [16,43]. Therefore, in the presented study, we focus on the relative quality assessment of examined brain tissue homogenization buffers.

Three different types of homogenization buffers were selected for the study: buffer with detergent, buffer with chaotropic agents and buffer without detergent (Table 1). The measurement data obtained were processed qualitatively and quantitatively.

The DB buffer allowed the highest yield of protein extraction from brain tissue, which is in accordance with comparisons of extraction buffers for brain tissue reported by Shevczenko et al. [43]. In both studies the protein concentration determined in the buffer with detergent (containing 1% SDS) was approximately three times higher compared to detergent-free buffer. However, for DB buffer the number of protein identifications was the lowest (Figure 1A–C). Although statistical analysis revealed significance, differences did not exceed 10% which was in line with results reported previously [43]. Even though the same tissue was analysed, and similar number of proteins were identified, about 10% of the proteins were buffer specific. This allowed for the clear sample grouping in principal component analysis (Figure 1D). The same intergroup relationships as for proteins were observed for number of peptides and unique peptides (Figure 1E,F). Gene ontology analysis showed that DB and CAB buffers provided more efficient extraction of neurorelevant proteins, while the DFB buffer might be more suitable for extraction of cytosolic proteins (Table 2).

Qualitative analysis did not reveal large differences between the buffers used (number of proteins and peptides identified, GO analysis) that would clearly support the selection of an optimal homogenization buffer for brain-tissue protein extraction in neurodegenerative disease research. Only quantitative analysis provided information on proteins belonging to important classes from the point of view of nervous system function, which made it possible to differentiate buffers in terms of suitability for brain-tissue analysis. It was determined that the DB buffer allows for a more efficient extraction of membrane, synaptic, and synaptic membrane proteins compared to CAB and DFB buffers, which was expected due to the chemical nature of the SDS molecule having the ability to extract more efficiently water-insoluble hydrophobic compounds. This observation is consistent with earlier reports by Wisniewski et al. that the use of SDS provides the highest representation of membrane proteins relative to others [10]. Also, the results reported by Ericsson et al. supports the recommendation to use the SDS for efficient extraction and solubilization of proteins from brain tissue [44]. As shown by MCL clustering, the DB buffer was the most suitable for the extraction of ribosomal proteins and myelin sheath proteins, the groups of proteins especially important in neurological diseases [45,46]. Both DB and CAB buffers were efficient in extracting mitochondrial proteins as well as pre- and post-synaptic proteins [47]. Since the CAB buffer contained urea and thiourea which are small molecules with the ability to interact with both polar and non-polar parts of proteins, it was not unexpected that the CAB buffer supported efficient extraction of the hydrophobic membrane proteins compared to the DFB buffer. In addition to previously published data, our study showed that the DFB buffer, which does not contain a detergent and a chaotropic agent, is slightly more efficient in the extraction of cytosolic proteins.

## 5. Conclusions

Based on our results, the detergent-based DB buffer is the most suitable for global differential proteomic profiling of brain tissue, especially in the context of neurodegenerative diseases (extracted protein clusters: mitochondrial, ribosomal, myelin sheath, membrane, synaptic and synaptic membrane). The CAB buffer allowed for obtaining the highest number of protein and peptide identifications, but label-free quantitative analysis determined it to be not the most suitable for quantitative analysis of brain tissue. The DFB buffer was determined to be the best for extraction of cytosolic and proteasomal proteins.

## Figures and Tables

**Table 1:** Composition of homogenization buffers used in the study.

Detergent Buffer (DB)	Chaotropic Agent Buffer (CAB)	Detergent-Free Buffer (DFB)
1% SDS 100 mM TEAB, pH 8.5 protease and phosphatase inhibitors	8 M urea, 2 M thiourea 50 mM Tris-HCl, pH 8.5 protease and phosphatase inhibitors	250 mM sucrose 150 mM NaCl 1 mM EDTA 50 mM HEPES, pH 7.0 protease and phosphatase inhibitors

**Table 2:** Number of proteins, divided in GO categories, identified in each type of homogenate.

	Detergent Buffer (DB)	Chaotropic Agent Buffer (CAB)	Detergent-Free Buffer (DFB)
membrane proteins	773	843	729
synaptic proteins	434	454	416
synaptic membrane proteins	90	100	77
cytosolic proteins	600	641	662

**Table 3:** Number of protein groups of interest for which different levels were found between each pair of homogenates.

	DB, CAB	DB, DFB	CAB, DFB
membrane proteins	64; 16	301;112	238; 103
synaptic proteins	29; 4	170; 50	129; 49
synaptic membrane proteins	4; 0	45; 5	41; 3
cytosolic proteins	43; 16	137; 198	95; 171

## Data Availability

The data presented in this study are available on request from the corresponding author.
